# Supplementary material for: Acceptance and compliance with micronutrient powder (MNP) among children aged 6–23 months in northern Nigeria
Source: PLOS Glob Public Health. 2022 Oct 17;2(10):e0000961. doi: 10.1371/journal.pgph.0000961 (PMC10022258; doi:10.1371/journal.pgph.0000961)
Supplement: S1 Dataset — (ZIP) [file pgph.0000961.s008.zip › S8_Dataset/In-depth Interviews.zip!/In-depth Interviews/030715_A_SO_PM_2_CG.pdf]

IDI WITH FEMALE CAREGIVER IN BONGO GARI COMMUNITY IN SONG LOCAL GOVT.

Q:Describe for me the people in your immediate family living in your household?

R:We are seven in number.4 females and 3 males.

Q:CAN YOU TELL ME THE AGES AND GENDER OF YOUR CHLDREN WHO ARE 6-23MONTHS?

R:There is idaru-a male-he is 22months old.there is abdul-a male-he is 4months old.

Q:WE HAVE HEARED OF FOOD CHALLENGES FOR YOUNG CHILDREN IN THIS SETTING RELATED TO THE SEASONS.WHAT ARE THE COPING MECHANISMS DO YOU EMPLOY TO COMBAT THIS FOOD CHALLENGES?

R:you see when i first got married we had serious issues regarding food me and my husband ,but ever since i started given birth we overcame that problem....there is no time you will come to my house you will lack food to eat, either raw, or cooked one. i really thank God on that aspect.

Q:SO YOU MEAN YOU DONT HAVE ANY CHALLENGE(S) OF FOOD AS IT AFFECTS YOUR YOUNG CHILDREN?

R:None at all.

WHAT ABOUT DURING RAINING AND DRY SEASONS DO YOU STILL ENJOY FOOD SUFFICIENCY?

R:You see during dry season there is availability of different kind of food.....so i can cook whatever variety i feel like. But in raining season and because of farming periods there is a bit of scarcity of food.....so what we normally do is to minimise the ration and ingredients we normally use to prepare food so as not to experience want and hunger.

Q:SO HOW DOES THIS AFFECT INFANT AND YOUNG CHILD FEEDING?

R:Well...it affects them in the sense that they may not be able to eat the different variety of food they prefer...assuming they are suppose to drink kunu in the morning but because there is shortage of food they may have to eat the left over of tuwo of last nite...because we canot afford to play around with food that is very scarce to come-by. But as i told you earlier on, in my household presently, i have overcome all this food problems.

Q:ONE THING I HEARED IN OTHER INTERVIEWS THAT CAREGIVERS OFTEN GIVE TO THEIR CHILDREN 6-23MONTHS IS “TEA”CAN YOU PLEASE EXPLAIN WHAT TEA MEANS?

R:It means the combination of milk,bournvita or lipton,sugar, and warm water.But there is no NESCAFE....because that is for adult alone....when you mix this items together then “tea”is ready for the child.....but for me i never give my child tea....because always it has to be this

kunu of sorghum mix with either soya beans or groundnut.That is why there is no time you come to my house that you will lack this flour of sorghum for kunu.

Q:NOW LETS TALK ABOUT THE MNP THAT YOU RECEIVED.TELL ME YOUR EXPREIENCE WITH THE PRODUCT?

R:Well...i was told of the importance of the MNP, but ever since i started applying for my child, i notice a lot of rashes on his body even on his head(skull),also, he has been stooling continuously...even up till today, thirdly, he always cry and he sleeps a lot....am a bit confused..

Q:So what have you done as prevention?

R:When i noticed the stooling, i went to the chemist to get him flygeen tablets....but it never worked....the stooling keeps coming out,the rashes is even my main concern because it is so plenty on his body.

Q:DID YOU EVER CONTEMPLATE DISCONTINUING THE USAGE OF THIS MNP BASED ON THE CHALLENGES YOU JUST ENUMERATED?

R:NO...i have never thought about that, because even the lady(ANTIYA)that monitored my child told me that such symptoms are normal, but i never thought it will last a long time like this.

Q:IS THERE ANY POSITIVE ASPECT OF THIS MNP?

R:The only thing i notice is that my child has increased in size. that is the only thing i noticed.

Q:SOME CAREGIVERS MENTIONED THAT IT WAS HARD TO STORE THE MNP WITHOUT IT BEING TAKEN BY OTHERS OR EATEN BY RODENTS.DID YOU HAVEV ANY SIMILAR EXPERIENCE?

R:No i don’t. I keep the mnp at the right place, nobody tampers with it, and i don’t have rodents in my house....except that attimes my other children could throw it(MNP) on the floor or on the bed in the process of trying to tidy the house.

Q:SOME MOTHERS INDICATED THAT IT MIGHT BE HARD TO REMEMBER TO USE MNP.DID YOU HAVE A SIMILAR EXPERIENCE?AND WHAT DID YOU DO TO REMEMBER?

Q:NO. i have never forgot to give him the MNP even for once, i was told of the importance of adhering to the usage of the product and its benefits to my child why would i forget such an important assignment? The only thing is that, sometimes, i might not be able to give him in the morning except in the evening or in the afternoon....but i have never failed to administer the powder to him.

Q:TELL ME ABOUT HOW YOU FEED THE MNP TO THE CHILD USUALLY?

R:Like i told you i normally mix the mnp in kunu,then i will mix it and give it to the child.

Q:IS IT ONLY KUNU THAT YOU HAVE BEEN USING?

R:That is what i was told.....that it is only kunu i can use.

Q:WHAT SORT OF KUNU IS THAT?

R:It is kunu dawa(sorghum)

Q:TO WHAT EXTENT DOES YOUR CHILD LIKES OR DISLIKES THE TASTE OF THIS MNP?

R:Well he is neutral about it....by that i mean he shows no reaction towards it....once i mix the mnp into his kunu he consumes it without stress....but at times you have to force him to drink, not because he does not like the taste but because of too much play.

Q;HAS THE CONSUMPTION OF THIS MNP INCREASED OR DECREASED THE CONSUMPTION RATE OF YOUR CHILD?

R:It has remain the same.

Q:IN OTHER COMMMUNITIES WE NOTICED THAT THE SHARING OF MNP OCCURED.COULD YOU TALK ABOUT ANY SHARING OF THE MNP WHEN YOU USED IT?

R:No i don’t think there is any form of sharing around here or in my household. l am very very strict with the usage of this mnp, i have already warned all of my other children with tampering with the powder......they would not even dare it....i can bring the mnp so you can count or check it by yourself.

Q:DID YOUR HUSBAND EVER USED THE MNP ON HIS OWN?

R:No he has never....the only he did was to taste the mnp in the kunu that was to be given to the child....that was all.

Q:AND WHAT WAS IS REACTION.....

R:I don’t think there was any reaction from him, because even me myself i have tasted the mnp once to feel how it taste like and i notice it is tasteless.

Q:HOW DO YOU HANDLE THE LEFT OVER OF FOOD THAT IS MIXED WITH THE MNP?

R:What i normally do is to prepare a moderate quantity of the kunu that the child can consume at once....then i will mix the powder, then give it to him to drink.....because that was the instruction given to me.....except that you know little children, at times he might finished the kunu at once, so what i normally do is to carry the leftover and keep it safely for him to consume later..

Q:AND DOES HE CONSUME IT?

R:Sometimes he refuses and other times he takes it.

Q:DO YOU THINK 60 SATCHETS OF THIS MNP WILL BE SUFFICIENT FOR 60 DAYS?

R:Well me am not a doctor....i can really say anything on that.....but i believe those that produce this mnp would no better. my own is to use as directed....if today they say i should be given the child two-two per day i will have no problem on that....so long as my child will be healthy and intelligent.

Q:HOW DO YOU PERCEIVE THIS MNP?DO YOU SEE AS A FOOD OR AS A MEDICINE OR SOMETHING ELSE?

R:In my own little local understanding i perceived it as food.

Q:WHY DO SAY SO?

R:Anything that is given to a child to consume is automatically a food....that is what i think.

Q:IF THIS MNP WERE TO BE SOLD VERY CHEAPLY IN YOUR COMMUNITY.WOULD YOU BUY IT?

R:Did you just said sold? because i was told that it will be given for free.

Q:AM ASSUMING IF IT WERE TO BE SOLD.

R:Well for now i can really place a price on it......but whatever is agreed upon is fine by me. moreover i don’t the intentions of the manufacturer....but by large for me i will definitely buy the powder....because as i was told it will increase the level of intelligence of a child aside other benefits.....that alone is a motivating factor for me to buy the product.

Q:CAN YOU PLEASE TELL ME SOME WAYS THAT YOU BELIEVE WILL BE EFFECTIVE IN PROMOTING THIS MNP TO CAREGIVERS IN THIS COMMUNITY SO THAT THEY WOULD COMPLY WITH USING IT APPROPRIATELY?

R:I think the involvement of our community leaders and health workers would be most valuable. for example...the community leader in collaboration with his advisers can decide to summon all the women for a particular health talk on how to effectively use this powder....this health talk should be given by the community health personnel since they are more experienced and familiar with most of the things.

Q:WHAT PEOPLE MOST INFLUENCE YOUR OPINIONS AND KNOWLEDGE ABOUT FEEDING PRACTICES?

R:Well we all live in a community where we have to interact and learn from each other...if you don’t understand you can easily ask or observe the ways other women are doing then,you

can quickly adjust if need be. what am trying to say is that we learn from each other(other older women)

Q:WHO INFLUENCES YOUR DECISIONS REGARDING FEEDING YOUR BABY WITH THE MNP?

R:It is those health workers that came around, also i was very pleased with the explanations and encouragements i received from the lady (ANTIYA) that monitored my child(DIRECT OBSERVATION)

Q:WHERE WOULD BE GOOD PLACES TO DISSEMINATE HEALTH INFORMATION RELATED TO MNP?

R:To my own understanding the community clinic would be most effective, because there is no single woman that does not no the location of this clinic, moreover the clinic will be more conducive and appropriate since most women are used to going there for health related issues especially as its affects infants.

Q:WHO IN YOUR COMMUNITY DO YOU TRUST THE MOST FOR RECEIVING MESSAGES ABOUT INFANT AND YOUNG CHILD FEEDING?

R:There is this my sister –in –law by the name BILKY, she lives in this community, but she is well educated, she knows most things relating to health, in short she normally discourage me from using anything that has to with traditional or herbal medicines....once any of my children is sick she will insist that the hospital will be the most reliable place to go.

Q:THIS YOUR SISTER-IN-LAW IS SHE A HEALTH WORKER?

R:No. BILKY is a graduate. she is through with her studies but she is yet to secure employment.

Q:WHAT FORMS OF MEDIA DO YOU PREFER FOR RECEIVING MNP MESSAGES

R:I feel if anything that has to do with health the local chiefs and his ward heads would be most effective. they can equally use the town crier to go round and make public announcements. At least almost everybody will hear the information.

Q:WHAT ABOUT POSTERS/FLYERS/AND BROCHURE?

R:How many women can read and write very well.....except you want to deceive yourself.

Q:WHAT ABOUT RADIO?

R:Is it everybody that owns a radio? me for example does not have a radio, so how would i receive such vital information. Except may be those fulanis in the bush....but even them is not all of them that are too keen with the radio, they prefer to spend most of their time tending after their flocks.

Q:WHAT ABOUT WORD OF MOUTH AND FROM WHOM?

R:Well, that is a bit better, but the implication is that there may be too many misinterpretations on the subject matter....everyone will try to interpret the issue the way he or she understands and they may pose a serious challenge.

Q:SO SPECIFICALLY WHAT WAYS OF RECEIVING EDUCATION REGARDING IYCF AND MNP DO YOU PREFER?

R:Like i said earlier, no better place than the community clinic. Everybody knows the place. Even people in the hinterland would require little or no description. most women, though not all do go for ante-natal, so to an extent most people are aware and are used to the clinic.

Q:CAN YOU REMEMBER ANY HEALTH EDUCATION CAMPAIGNS IN THE PAST IN THIS COMMUNITY THAT WAS VERY SUCCESSFUL?

R:None that i can remember. Aside the issue of immunization that once in a while they will announce through the town crier that women and even sometimes the men should assemble at the clinic to receive a particular vaccination....and even at that it is not everybody that normally turns out....but still you will witness a large turn out of people...this is the only thing i can remember.

Q:SO ONCE MORE,WHAT BETTER WAYS OR CHANNELS DO YOU FEEL WOULD BE MOST APPROPRIATE TO DISTRIBUTE THIS MNP AND WHY?

R:I have already told you....that we either use the clinic and the health workers or we involved the community leader. But personally the health workers in the clinic would be more appropriate because of their expertise and experience in dealing with this kind of issues. why i am not 100% in support of the village chiefs is that they may politicise the whole campaign or they may be sentimental in distributing the products.....but they are still very important people if you really wants to have a large turn out of people.

Q:CAN YOU TELL ME SOME SPECIFIC MESSAGES YOU THINK WILL EFFECTIVELY PROMOTE THIS PRODUCT?

R:We can use words like “immune booster” or “intelligent provider “ to qualify the products and to convince most women to key in into the programme. Because no woman who knows that a product would boost her child immunity or increase his level of intelligence and will play with such a product. i think if you can explain properly to most women the health benefits associated with this products i think most women even the husbands would welcome it.

Q:WHAT ARE THE EFFECTIVE MESSAGES YOU THINK CAN BE USE TO PROMOTE THE USE OF THIS PRODUCTS BUT LIMITS THE SHARING OF ITS?

R:You see there is nothing as important than to sound a note of WARNING OR TO READ OUT THE IMPLICATIONS OF SHARING THIS PRODUCTS AMONG EACH OTHER OR AMONG THE CHILDREN. You see a village person is always afraid of things that will puts him or her into trouble so they always try to run away from such. moreover if there can

be regular monitoring by EXTERNAL PEOPLE on the appropriate usage of this products by caregivers that will also go a long way in putting people on their toes to do the right thing.

Q:FINALLY,IS THERE ANYTHING ELSE THAT YOU CAN SHARE ABOUT THIS PRODUCT...LIKE PLACEMENT,PRICE OR PROMOTION.....

R:well for the price, if it were possible let its(the MNP) be free of charge if really you want people to fully participate on this programme, because once you place a price you will automatically disenfranchise a lot of people from partaking. look common to go the clinic and pay for #50 card is a big problem to most people talk more of paying for something that will not directly benefits them.

Q:WHAT ABOUT IN TERMS OF PROMOTING THE PRODUCT?

R:I think i have already explain that to you.

Q:OKAY THANK YOU...I JUST NEEDED TO BE SURE THAT WE ARE FLOWING TOGETHER.IS THERE ANY OTHER THING YOU CAN RECCOMEND IN OTHER TO DEVELOP AN EFFECTIVE MNP PROGRAMME.

R:Me i am ordinary petty trader. you can meet those staff in the clinic they will help you with some of this suggestion because i believe they have previous experience on how to go about this kind of programme.

Q:I KNOW ABOUT THIS HEALTH WORKERS, BUT IT IS YOUR OWN PERSONAL VIEW THAT I AM INTERSTED IN

R:I have already told you the little i know.

Q:okay thank you very much for your time......i have a few more questions to ask you. i would not use this recorder, i would rather write out your responses. thank you once more.
